# The Effects of Responsible Gambling Pop-Up Messages on Gambling Behaviors and Cognitions: A Systematic Review and Meta-Analysis

**DOI:** 10.3389/fpsyt.2020.601800

**Published:** 2021-01-25

**Authors:** Benjamin Bjørseth, Josefine Oudmayer Simensen, Aina Bjørnethun, Mark D. Griffiths, Eilin K. Erevik, Tony Leino, Ståle Pallesen

**Affiliations:** ^1^Department of Psychosocial Science, University of Bergen, Bergen, Norway; ^2^International Gaming Research Unit, Psychology Department, Nottingham Trent University, Nottingham, United Kingdom; ^3^Norwegian Competence Center for Gambling and Gaming Research, University of Bergen, Bergen, Norway; ^4^Optentia, The Vaal Triangle Campus of the North-West University, Vanderbijlpark, South Africa

**Keywords:** gambling, responsible gambling, gambling behavior, gambling cognition, pop-up message, warning message, meta-analysis, dynamic warning message

## Abstract

Pop-up messages utilized by gambling operators are normally presented to gamblers during gambling sessions in order to prevent excessive gambling and/or to help in the appraisal of maladaptive gambling cognitions. However, the effect of such messages on gambling behavior and gambling cognitions has not previously been synthesized quantitatively. Consequently, a meta-analysis estimating the efficacy of pop-up messages on gambling behavior and cognitions was conducted. A systematic literature search with no time constraints was performed on Web of Science, PsychInfo, Medline, PsychNET, and the Cochrane Library. Search terms included “gambling,” “pop-up,” “reminder,” “warning message,” and “dynamic message.” Studies based on randomized controlled trials, quasi-experimental designs and pre-post studies reporting both pre- and post-pop-up data were included. Two authors independently extracted data using pre-defined fields including quality assessment. A total of 18 studies were included and data were synthesized using a random effects model estimating Hedges'* g*. The effects of pop-ups were *g* = 0.413 for cognitive measures (95% CI = 0.115–0.707) and *g* = 0.505 for behavioral measures (95% CI = 0.256–0.746). For both outcomes there was significant between-study heterogeneity which could not be explained by setting (laboratory vs. naturalistic) or sample (gambler vs. non-gamblers). It is concluded that pop-up messages provide moderate effects on gambling behavior and cognitions in the short-term and that such messages play an important role in the gambling operators' portfolio of responsible gambling tools.

## Introduction

Gambling can be defined as wagering money or other objects of value on an event of an uncertain outcome that is partly or completely determined by chance (1), and has become increasingly available to individuals due to such factors as increased accessibility via the internet and liberalization of gambling regulation. For most individuals, gambling represents a recreational activity. However, it is estimated that between 0.1 and 3.4% of the population in Europe and 0.1–5.8% of the population worldwide engage in problematic gambling behavior (2). Problem gambling is, according to Blaszczynski et al. [(3), p. 305], “a lay term that refers to a broader category of individuals exhibiting patterns of excessive gambling behavior that is associated with harmful effects” (p. 305). The terms “pathological gambling” and “gambling disorder” refer to a more specific pattern of problematic gambling, and has been classified as a mental disorder (3). Blaszczynski et al. [(3), p. 305] suggest that “problem gamblers may or may not suffer impaired control” and “conceptually, all pathological gamblers are problem gamblers, but not all problem gamblers are pathological gamblers.”

Gambling disorder (formerly pathological gambling) was the first non-chemical (i.e., behavioral) addiction to be recognized in formal diagnostic systems (4). According to the fifth edition of *Diagnostic and Statistical Manual of Mental Disorders* (DSM-5), gambling disorder is defined by nine criteria similar to those of substance abuse, such as lack of control, tolerance, withdrawal, and the maintaining of harmful behavior despite negative consequences (5). In the eleventh revision of the *International Statistical Classification of Diseases* (ICD-11), the World Health Organization (6) defines gambling disorder as “a pattern of persistent or recurrent gambling behavior, which may be online (i.e., over the internet) or offline.” The gambling behavior of individuals suffering from gambling disorder can significantly affect an individual's personal, family and social life, as well as their educational and/or occupational functioning (7–9).

Treatment of gambling disorder is associated with involvement of health services (e.g., counseling administered by specialists employed in public health programs). An important feature of treatment is the emphasis on gamblers who are already suffering from severe gambling problems (3). Different treatment approaches have been developed, including cognitive behavioral therapy incorporating motivational interviewing (10), mindfulness (11), and pharmacological treatment (12, 13). Overall, results show positive effect of these treatments. However, limitations across studies points to a lack of evidence of successful long-term effects and attrition (10–14). Furthermore, the treatment of problem gambling is usually costly and few of those affected seeks out treatment on their own (15, 16). For these reasons, the development and implementation of effective and cost efficient tools to reduce gambling-related problems seems warranted.

The past two decades' efforts to create, shape, and implement responsible gambling (RG) strategies and programs regarding the management of gambling-related activities, have been characterized by two main frameworks [(17), p. 1]: The Public Health Model and the Reno Model, with the latter historically and geographically being the more influential of the two (18). While it is true that they share some objectives (e.g., reliance on strong empirical data and collaborative efforts between stakeholders), they differ in the areas of focus and approaches (17, 19–21).

One of the central tenets of the Reno model, is informed choice—the making of a decision based on as much information as possible—“the ultimate decision to gamble or not lies with the gambler” [(17), p. 9]. Furthermore, the Reno Model states that measures to prevent problem gambling should be as non-intrusive as possible, so as to let recreational gamblers engage in “healthy gambling” (i.e., gambling without negative/adverse consequences). It also stresses the point that efforts from the medical community (i.e., treatments and prevention) ought to specifically target at-risk groups, without being intrusive toward the larger population (22). The Reno Model's emphasis on individual responsibility, informed choice, personal control, and prioritization of the recreational benefits of gambling, has been critiqued by Hancock and Smith (20), Delfabbro and King (19), and Young and Markham (21) for being ideological at its core with libertarianism as its central tenet. Hancock and Smith (18) further criticize the Reno Model for its minimal regard to effective RG safeguards following the last two decades' rapid increase of gambling.

The Public Health Model, casting a wider net, seeks the widespread use of epidemiological studies to map the impact of gambling-related harm (23). Public health officials should, in line with this perspective, view gambling as a population-based phenomenon and seek to identify the cultural, social, and economic factors that mediate gambling (17). Public policy should be guided in such a way so as to increase health in the population and to prevent gambling-related harm (23). The Public Health Model suggests implementing guidelines that promote healthy gambling, including large-scale public informational campaigns, similar to informational campaigns regarding alcohol and tobacco, about the possible adverse effects of gambling (23). The Public Health Model further urges for public policy and governmental legislation to regulate gamblers' behavior in such a way so as to reduce the likelihood of gambling-related harm (e.g., mandatory loss-limits, mandatory breaks in play, and reduced accessibility during specific hours) (23). Hancock and Smith (20), and Young and Markham (21) have called for a broad implementation of the Public Health Model on account of what they argue is the failure of the Reno Model to properly implement measures to reduce gambling-related harm.

“Responsible gambling” can be defined as the “policies and practices designed to prevent and reduce potential harms associated with gambling” and is emphasized by both frameworks [(3), p. 308]. More explicitly, RG refers to guidelines, strategies, or programs that attempt to avert possible harmful health outcomes, in contrast to the principles of treatment (3). RG may also imply “means to prevent gambling problems or to reduce the negative consequences of existing problems” [(24), p. 1,376]. “Means” may refer to “self-exclusion programs; behavioral tracking of play patterns; loss and deposit limit setting (both player and corporate); player pre-commitment to deposits, losses, wins, or gambling time; and warning messages” [(25), p. 225]. Most of these measures require registration and collection of player account gambling data, which are enabled by gambling via online gambling accounts or via loyalty card/player cards (26). An example of RG measures put into practice, is New Zealand's policy regarding EGMs in gambling venues, which are required by law to display pop-up messages to interrupt play at “irregular intervals not exceeding 30 min of continuous play” [(27), p. 1,116].

Hence, RG is about the necessity to sustain a safe environment for gamblers, and because the main objective of RG programs is to prevent gambling related harm, “[RG] programs should provide information that consumers use to make decisions” [(28), p. 570], which represents a perspective on RG tools congruent with the Reno Model. The use of pre-commitment to limit expenditure is an example of this. Such a measure permits gamblers to regulate how much money and/or time they can spend gambling, and allows gamblers to temporarily (or permanently) exclude themselves from gambling (24). Personal feedback interventions (PFIs) are another approach, where information about an individual's gambling behavior is compared to another individual's gambling behavior, and then presented to the gambler (29).

Pop-ups or dynamic messages, as understood in an RG context, comprise informative messages appearing on screen during gambling, halting play, with the overall aim of preventing and/or reducing gambling-related harm (30). The message subsequently either disappears after a set duration of time or requires some kind of action to be taken (e.g., pressing “*OK”* or “*Press/click here to continue”*) on the part of the gambler [(31), p. 933]. It can be used to present factually descriptive or normative information, such as information about time and/or monetary expenditure, either regarding the individual player solely, or in comparison with other gamblers (30). It can also be used to correct irrational or distorted beliefs about gambling (e.g., “*Winning is not due to luck. It's random”* or “*The next spin has nothing to do with your previous spin”*) (31), or as a reminder of progress toward a previously set limit (i.e., progression toward a monetary limit set before initiating a gambling session) (30). Furthermore, messages can be worded to encourage self-appraisal, so as to increase gamblers' awareness of their own gambling behavior (e.g., “*Pause and think … Are you in control of your risk taking?”*) (32, 33). Pop-up messages can vary in time of appearance, and usually appear after a set duration of time or a set monetary expenditure (34). In short, pop-up messages serve as a tool to deliver RG information to gamblers during play.

Several researchers have investigated both the efficacy of information delivery and the effect of various types of content on expenditure and time spent playing (35–38). Furthermore, Monaghan and Blaszczynski (39) conducted a study on recollection of message content, where the participants recalled dynamic messages (pop-up messages) more easily than static messages, and another study where pop-up messages where recalled more effectively than static messages, both immediately and at a 2-weeks follow-up (33). Studies on pop-up messages and limit setting—that is, a monetary limit set by the gambler before initiating a gambling session—suggest that individuals exposed to monetary limit reminders via pop-up messages, are more likely to adhere to the pre-set limits (40, 41).

As previously mentioned, gambling is more accessible to the public than ever before, which in turn may increase problem gambling. This, combined with the fact that there is support to suggest that pop-up messages can be an effective RG tool, and that (to date) no meta-analysis have been conducted on the effects of pop-up messaging (as far as the authors of the present meta-analysis are aware of), the goal of the present meta-analysis is to explore the effect of pop-up messages on gambling-related behaviors and cognitions.

## Method

The present meta-analysis was conducted in accordance with the guidelines of the Preferred Reporting Items for Systematic Review and Meta-Analyses (PRISMA) (42, 43). For complete checklist, see Table 1.

**Table 1:** PRISMA checklist.

**Section/topic**	**#**	**Checklist item**	**Reported on page #**
**TITLE**			
Title	1	Identify the report as a systematic review, meta-analysis, or both.	1
**ABSTRACT**			
Structured summary	2	Provide a structured summary including, as applicable: background; objectives; data sources; study eligibility criteria, participants, and interventions; study appraisal and synthesis methods; results; limitations; conclusions and implications of key findings; systematic review registration number.	2
**INTRODUCTION**			
Rationale	3	Describe the rationale for the review in the context of what is already known.	3–5
Objectives	4	Provide an explicit statement of questions being addressed with reference to participants, interventions, comparisons, outcomes, and study design (PICOS).	5
**METHODS**			
Protocol and registration	5	Indicate if a review protocol exists, if and where it can be accessed (e.g., Web address), and, if available, provide registration information including registration number.	
Eligibility criteria	6	Specify study characteristics (e.g., PICOS, length of follow-up) and report characteristics (e.g., years considered, language, publication status) used as criteria for eligibility, giving rationale.	6-−7
Information sources	7	Describe all information sources (e.g., databases with dates of coverage, contact with study authors to identify additional studies) in the search and date last searched.	7
Search	8	Present full electronic search strategy for at least one database, including any limits used, such that it could be repeated.	7
Study selection	9	State the process for selecting studies (i.e., screening, eligibility, included in systematic review, and, if applicable, included in the meta-analysis).	7
Data collection process	10	Describe method of data extraction from reports (e.g., piloted forms, independently, in duplicate) and any processes for obtaining and confirming data from investigators.	7
Data items	11	List and define all variables for which data were sought (e.g., PICOS, funding sources) and any assumptions and simplifications made.	6
Risk of bias in individual studies	12	Describe methods used for assessing risk of bias of individual studies (including specification of whether this was done at the study or outcome level), and how this information is to be used in any data synthesis.	8
Summary measures	13	State the principal summary measures (e.g., risk ratio, difference in means).	8
Synthesis of results	14	Describe the methods of handling data and combining results of studies, if done, including measures of consistency (e.g., I^2^) for each meta-analysis.	8
Risk of bias across studies	15	Specify any assessment of risk of bias that may affect the cumulative evidence (e.g., publication bias, selective reporting within studies).	9
Additional analyses	16	Describe methods of additional analyses (e.g., sensitivity or subgroup analyses, meta-regression), if done, indicating which were pre-specified.	8
**RESULTS**			
Study selection	17	Give numbers of studies screened, assessed for eligibility, and included in the review, with reasons for exclusions at each stage, ideally with a flow diagram.	9
Study characteristics	18	For each study, present characteristics for which data were extracted (e.g., study size, PICOS, follow-up period) and provide the citations.	10
Risk of bias within studies	19	Present data on risk of bias of each study and, if available, any outcome level assessment (see item 12).	25
Results of individual studies	20	For all outcomes considered (benefits or harms), present, for each study: (a) simple summary data for each intervention group (b) effect estimates and confidence intervals, ideally with a forest plot.	Figures 2, 4
Synthesis of results	21	Present results of each meta-analysis done, including confidence intervals and measures of consistency.	11
Risk of bias across studies	22	Present results of any assessment of risk of bias across studies (see Item 15).	10–11
Additional analysis	23	Give results of additional analyses, if done (e.g., sensitivity or subgroup analyses, meta-regression [see Item 16]).	11
**DISCUSSION**			
Summary of evidence	24	Summarize the main findings including the strength of evidence for each main outcome; consider their relevance to key groups (e.g., healthcare providers, users, and policy makers).	12–14
Limitations	25	Discuss limitations at study and outcome level (e.g., risk of bias), and at review-level (e.g., incomplete retrieval of identified research, reporting bias).	13
Conclusions	26	Provide a general interpretation of the results in the context of other evidence, and implications for future research.	12-13
**FUNDING**			
Funding	27	Describe sources of funding for the systematic review and other support (e.g., supply of data); role of funders for the systematic review.	14

### Eligibility Criteria: Participants, Interventions, Comparators

The meta-analysis included (i) randomized controlled trials, quasi-experimental studies as well as pre-post studies investigating the effect of (ii) RG pop-up messages on (iii) gambling behaviors and/or gambling cognitions in (iv) gamblers and non-gamblers of (v) all ages equal to or above the legal gambling age, and (vi) published in peer reviewed journals or as conference presentations.

A study was deemed to include a comparator/control group if the intervention group was compared to a group of any of the following kind: (i) no pop-up message control (including cases with other forms of warnings or pauses before, during, or after play), (ii) passive pop-up message controls (e.g., “click ok to continue”), (iii) irrelevant pop-up message intervention (e.g., “*The roulette game was invented in 1720”*), or (iv) active pop-up messages assumed to have significantly less of an effect than the experiment intervention (e.g., “*You have now played 1,000 slot games. Do you want to continue? (YES/NO)”* vs. “*We would like to inform you, that you have just played 1,000 slot games. Only a few people play more than 1,000 slot games. The chance of winning does not increase with the duration of the session. Taking a break often helps, and you can choose the duration of the break”*) [(36), p. 3]. In studies containing two or more control conditions, the control group selected for effect size calculations was chosen in line with the aforementioned order [e.g., (44)].

Studies with a quasi-experimental design (e.g., studies without randomization of participants to conditions) were included as this is often the only method of getting real life data from gambling providers. Furthermore, studies lacking comparison/control conditions were included if both pre- and post-intervention data were reported or obtained from the author(s).

RG pop-up messages were operationalized as dynamic messages intended to reduce gambling harm in some form or another, by interrupting play and that provided either (i) information regarding gambling behavior (e.g., time spent, trials played, money spent, progression toward a pre-set limit), (ii) general information about the nature of gambling machines (e.g., “*You cannot predict anything in a game of chance”*), or (iii) messages containing encouraging self-appraisal (e.g., “*Stop and think… Are you in control of your risk-taking?”*). Furthermore, RG pop-up messages had to appear on a gambling device containing a screen [e.g., electronic gaming machine [EGM] or personal computer]. This implied that studies where pop-ups either appeared prior to gambling or after gambling had ended, were administered via other forms of communications (e.g., email or SMS), or randomly over a longer period (i.e., weeks or months), were excluded [e.g., (45)]. In addition, this also meant that studies with encouraging messages (e.g., “*You are a skillful player!”*) were excluded, as were studies where RG pop-ups were not the primary independent variable (e.g., the message “*The game is now paused,”* followed by a forced pause in the game). Outcome measures of interest were pooled into two main categories: (i) behavioral (e.g., total number of bets, total amount spent, limit adherence), and (ii) cognitive (e.g., recall, arousal, dissociation).

The population in all included studies were classified as either gamblers or non-gamblers. This categorization was made by the authors of the individual included studies. Trials contained participants of both sexes and all ages, as long as they were equal to or over the legal age to gamble (this was 18 years in most cases, but could be 20 or 21, depending on area of jurisdiction). The trials took place in one of the following locations: (i) gambling venue, (ii) laboratory, and (iii) online. Studies were excluded if they (i) failed to meet the aforementioned inclusion criteria, (ii) were duplicates, (iii) written in a language other than Norwegian, Swedish, Danish, English, or Italian, (iv) did not contain sufficient information to calculate effect size, or (v) had self-report data on behavioral measures (e.g., time spent, amount spent, number of spins).

### Search Strategy

Studies were identified by searching electronic databases, reference lists of relevant papers, and through contacting study authors, in cases where supplementary information was needed. No limits were set on language or time periods. The search was conducted on Web of Science (1945-Present), APA PsychINFO (Ovid) (1806-Present), Medline (Ovid) (1946-present), PubMed (1993-present), APA PsychNET (unable to retrieve information on time period), Cochrane Library on Wiley Online Library [including Cochrane Database of Systematic Reviews, Database of Abstracts of Reviews of Effects (DARE)] and the Cochrane Central Register of Controlled Trials (CENTRAL) (1995-present). The last search was conducted by the first author on May 21, 2020.

The following search terms were used in all databases and trial registers searched: “gambling”; “pop up”; “pop ups”; “pop-up”^*^; “reminder”; “warning message”; and “dynamic message.” The final search strategy was developed through identification and discussion of relevant keywords. Preliminary searches were conducted to identify further relevant keywords. The final search strategy was agreed upon through consensus. For detailed overview of search terms and search strategy, see Table 2.

**Table 2:** Search terms and strategy.

**Searches and databases**
Abbreviations: “ti”: title; “ab”: abstract; “kw”: keyword; “mp” in the APA PsycINFO database includes: title, abstract, heading word, table of contents, key concepts, original title, tests & measures, mesh; “any field” in the APA PsycINFO and Medline databases includes: author, journal title, book title, keywords, first page, title, abstract, affiliation, author of review item, conference, correction date, correspondence, DOI number, geographic location, grant/sponsorship, index terms, ISBN, ISSN, language, MeSH: medical subject heading, publication date, publisher, PubMed ID, release date, tests & measures, title of review item, unique identifier, year of review item; “all fields” in the PubMed database includes: affiliation, author, author – corporate, author – first, author – identifier, author—last, book, conflict of interest statements, date—completion, date—create, date—entry, date—mesh, date—modification, date—publication, EC/RN number, editor, filter, grant number, ISBN, investigator, issue, journal, language, location ID, mesh major topic, mesh subheading, mesh terms, other term, pagination, pharmacological action, publication type, publisher, secondary source ID, subject—personal name, supplementary concept, text word, title, title/abstract, transliterated title, volume; “all” in the Web of Science database includes: topic, title, author, author identifiers, group author, editor, publication name, DOI, year published, address, organization-enhanced, organization, suborganization, abstract, author keywords, keyword plus, street address, city, province/state, country/region, zip/postal code, funding agency, grant number, funding text, research area, web of science category, ISSN/ISBN, accession number, PubMed ID.
**APA PsycINFO (Ovid)**
gambling AND (“pop up” OR “pop ups” OR pop-up^*^ OR reminder OR “Warning message^*^” OR “Dynamic message^*^”): mp
**APA PsycNet**
gambling AND (“pop up” OR “pop ups” OR pop-up^*^ OR reminder OR “Warning message^*^” OR “Dynamic message^*^”): any field
**Cochrane Library**
gambling AND (“pop up” OR “pop ups” OR pop-up^*^ OR reminder OR “Warning message^*^” OR “Dynamic message^*^”): ti,ab,kw (Word variations have been searched)
**MEDLINE (Ovid)**
gambling AND (“pop up” OR “pop ups” OR pop-up^*^ OR reminder OR “Warning message^*^” OR “Dynamic message^*^”): mp
**PubMed**
gambling AND (“pop up” OR “pop ups” OR pop-up^*^ OR reminder OR “Warning message^*^” OR “Dynamic message^*^”): all fields
**Web of science**
gambling AND (“pop up” OR “pop ups” OR pop-up^*^ OR reminder OR “Warning message^*^” OR “Dynamic message^*^”): all

### Data Extraction

All studies were screened independently by two authors. Most studies were excluded based on screening of title and abstract (e.g., when it was apparent that the study did not report on the effects of pop-ups). Inclusion to the next review stage was determined by consensus, and by consultation with a third author. Full texts were subsequently screened by two authors independently, with disagreements being resolved through discussion and consultation with the third author. The reference lists of all included studies in the meta-analyses were also screened.

A data extraction sheet, based on the Cochrane Consumers and Communication Review Group's data extraction template, was developed by two authors and pilot-tested on eight randomly selected studies. The extraction sheet was then refined to code further aspects of studies included. Data were extracted from the included studies by the three first authors and the extracted data then checked by two authors independently. Disagreements regarding extracted data were resolved through discussion.

In cases without sufficient data to calculate effect sizes, authors listed with contact information were contacted via email. Means, standard deviations, and sample sizes on all measures were obtained from four sets of authors (46–49) as data had only been presented graphically or was not included in the original publication. Furthermore, four authors (50–53) responded, but were for various reasons unable to provide data. Additionally, three authors did not respond.

Data were extracted from each trial on: (i) characteristics of participants (age, gender, and categorization into gamblers or non-gamblers); (ii) study design (including between-participants design, pre-post measurement design, and repeated measures design); (iii) exclusion and inclusion criteria; (iv) type of intervention, including the type of pop-up intervention and the type of control condition (i.e., no intervention, passive intervention, or intervention assumed less effective than experiment intervention); (v) type of outcome measure (behavioral and/or cognitive), and (vi) follow-up interventions. Behavioral outcome measures were defined as any type of gambling action measured, while cognitive measures comprised any type of gambling cognition assessed.

### Assessment of Risk of Bias

Risk of bias in individual studies was assessed using the Evidence Project Risk of Bias Tool developed by Kennedy et al. (54). Using this tool, two individual authors assessed the following areas of bias: (a) cohort, (b) control or comparison group, (c) pre-post intervention data, (d) random assignment of participants to the intervention, (e) random selection of participants for assessment, (f) follow-up rate of 80% or more, (g) comparison groups equivalent on socio-demographics, and (h) comparison groups equivalent on outcome measures at baseline. Items *a, b, c*, and *e* are dichotomous and have the response options “yes” and “no,” *d* is categorical and has “yes,” “no,” and “NA” (not applicable) as response options, whereas *f, g*, and *h* are categorical and have the response options “yes”, “no”, “NA” and “NR” (not reported) as response options. In cases of disagreement, a third author was consulted and disagreements resolved through discussion. Risk of bias was assessed at both the study and outcome level and Cohen's kappa was calculated to assess inter-rater reliability. No risk of bias scores were calculated, based on the recommendations of Kennedy et al. (54).

### Meta-Analyses

Two meta-analyses were conducted: one investigating cognitive measures, and the other investigating behavioral measures. Cognitive measures comprised different non-behavioral outcomes such as player experience, keeping track of play, estimation of time and money spent, erroneous beliefs, dissociation, and recall of pop-up messages, whereas the behavioral outcomes typically consisted of measures such as amount of money spent, speed of gambling, number of games played, etc. For each study, Hedges' *g* was computed. The primary outcome measure of the meta-analyses was Hedges' *g* and Cochrane's *Q*. *I*^2^ were calculated to assess heterogeneity, as it reflects the proportion of variation in observed effects that is due to variation in true effects (55). *I*^2^ values of 0.25, 0.50, and 0.75 are regarded as small, medium, and large, respectively (56). Initially, additional meta-analyses were planned to assess the effect at follow-up. However, in the sample of studies, only two included follow-up data (33, 53), which rendered meta-analyses for follow-up data less meaningful. Therefore, no additional meta-analyses were conducted. Furthermore, in cases where studies had more than one pop-up condition not conceptually different from each other, the groups were collapsed, and standard error—and consequently the 95% confidence intervals—were adjusted accordingly, in order not to repeat the control group data, as per the recommendations of Giang et al. (57).

In cases with more than one pop-up condition conceptually different from the other, the control group was split into corresponding numbers of groups, as recommended in the *Cochrane Handbook for Systematic Reviews of Interventions* (58).

Two moderators were decided upon in case of significant heterogeneity: (i) laboratory setting vs. naturalistic setting, and (ii) gamblers vs. non-gamblers (a sample was deemed to consist of non-gamblers in cases where they accounted for 50% or more of the total sample). Many studies included multiple measures within the same category (category referring to either behavioral or cognitive). In these instances, the mean effect size and variance was calculated for the study as a whole. When combining results from more than one outcome within the same outcome category from the same study, setting the correlation coefficient between outcomes to the default (r = 1.00) used in most meta-analytic software overestimates the standard error (59). To correct for this, the correlation coefficient between the outcomes was set to 0.70.

In cases of significant heterogeneity, subgroup analyses were conducted with a focus on the two aforementioned *a priori* determined moderators. The moderator analyses comprised mixed effects models (random across subgroups pooling tau across studies, but combining subgroups using fixed effect models), as recommended by Borenstein et al. (59). The planned moderator analyses were conducted when there were four or more studies within each category, in line with the suggested minimum criteria for number of studies (60).

### Publication Bias

Publication bias was examined by creating and inspecting funnel plots, and by using Duval and Tweedie's (61) “trim and fill” procedure, calculating a new and adjusted effect size which takes into account potential publication bias. In addition to this, Orwin's fail safe *N* was calculated, measuring the number of studies with zero effect needed to bring the observed effect size (Hedge's *g*) down to a pre-set trivial effect size (62), set to *g* = 0.20, which equals a small effect (63).

## Results

### Selection and Inclusion of Studies

**Figure 1:**
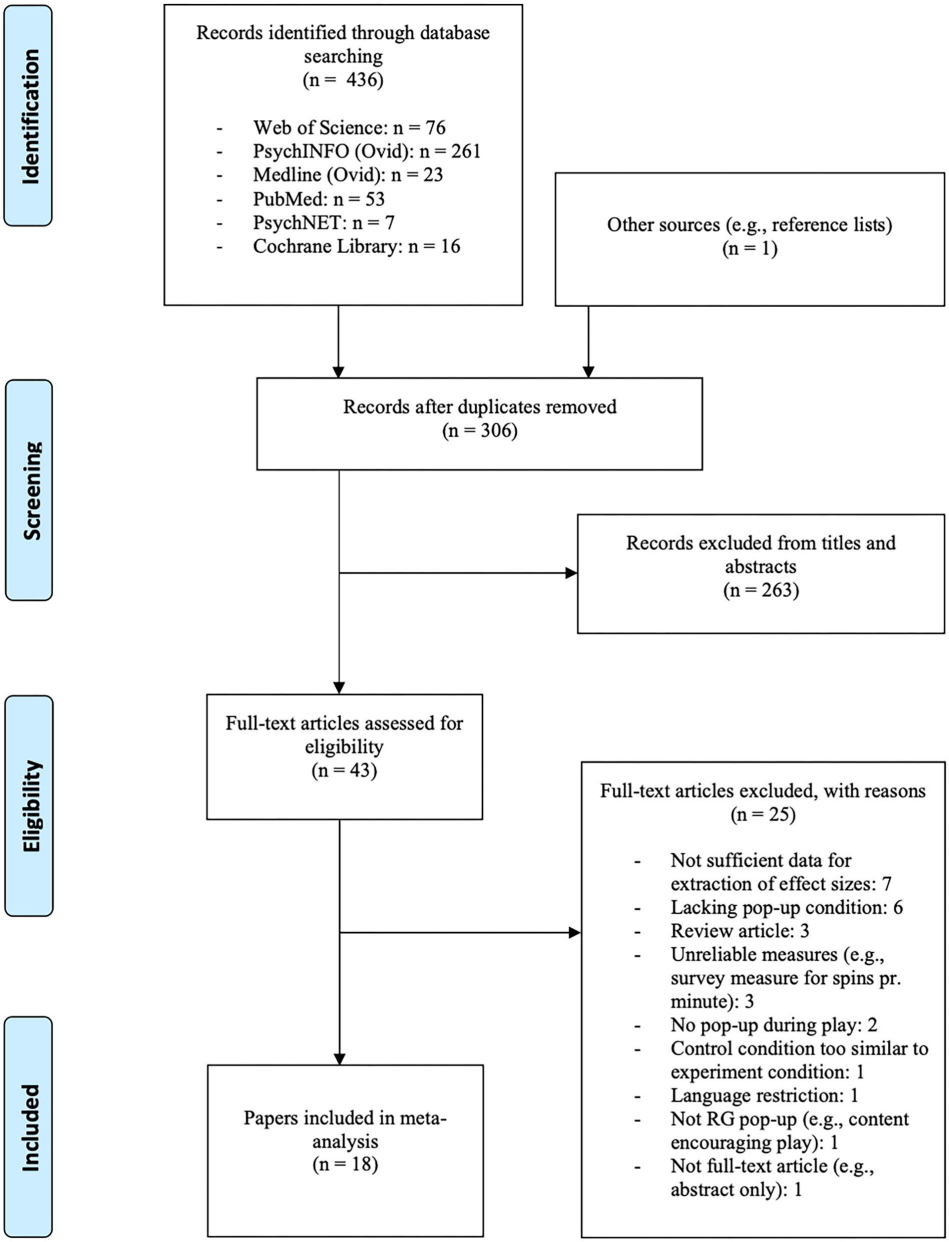
PRISMA flow chart.

A total of 18 papers involving 19 studies [(33) included two individual studies] were deemed eligible and were included in the present meta-analyses. The systematic searches conducted in Web of Science, PsychInfo, Medline, PubMed and Cochrane Library yielded a total of 436 hits. A total of 306 papers remained after the removal of duplicates. All of the remaining papers were systematically reviewed by the authors. Of the 306 papers, 263 were excluded based on title and abstract. This left 43 papers to be assessed for eligibility, of which 25 were excluded for the following reasons: seven did not provide sufficient data for calculation of effect size, six lacked a pop-up message condition altogether, three included measures deemed unreliable (e.g., self-report measures for spins per minute), three were review articles, two lacked a pop-up message condition during play, one had experiment and control condition deemed too similar, one contained a non RG-pop-up message (i.e., the message content was encouraging play), one was excluded due to language restrictions, and one was an abstract with no full text paper available. One unpublished study was identified through the database searches and found eligible. The screening process can be found in the PRISMA flow chart (see Figure 1).

### Characteristics of the Included Studies

In total 14 studies were conducted in laboratory settings, three were *in situ* (naturalistic) studies, and one study was conducted in a laboratory setting and subsequently replicated in a naturalistic setting. All studies were published in English. Furthermore, all studies were conducted within the past 15 years (from 2006 to 2019). Participants in seven out of the 18 studies were university students whereas participants in 12 out of the 18 studies consisted of non-university gamblers of all types. Nine of the included studies did not display pop-ups in their control condition, seven displayed some variation of a pop-up message in the control condition and two used pre-pop-up measures as the control condition. Only two studies included follow-up assessments, at 1 and 2 weeks, respectively. Eight studies contained outcome measures for behavior only and two for cognition only, whereas eight included outcomes for both behavior and cognition. The total number of participants included in the studies could not be assessed exactly because two studies listed approximate sample sizes of 50,000 [i.e., (35)] and 70,000 [i.e., (36)]. Studies lacking information on the socio-demographics of their samples were assumed to contain both genders and different ages, as the sheer size would have made gender and age homogeneity highly unlikely. Tables 3–5 provide a complete overview of the studies' key properties and characteristics.

**Table 3:** Study characteristics, studies A-H.

**References**	**Study design**	**Participants (N at start of trial in parenthesis)**	**Mean age**	**Sex**	**Inclusion criteria**	**Type of intervention in experimental condition**	**Type of intervention in control condition**	**Outcome**	**Measures**	**Follow-up**
Auer and Griffiths (36)	Naturalistic	Approximately 70.000 online slot machine gamblers	NR	NR	Gamblers on gambling site	Enhanced (normative and self-appraisal feedback) pop-ups	Non-enhanced pop-up	Behavior	Termination of session	None
Auer et al. (35)	Naturalistic	Approximately 200.000 gamblers	NR	NR	Gamblers on gambling site	Slot machine pop-up	Pre pop-up intervention	Behavior	Termination of session	None
Byrne et al. (46)	Lab/RCT	213	38.83	108 males; 104 females; 1 other	Gamblers of all EGM experience levels	Present pop-up message	No pop-up	Behavior and Cognition	Behavior: Gambling persistence, keeping track of play usage; Cognition: Estimation accuracy; player experience	None
Cloutier et al. (64)	Lab/RCT	40	22.20 (control group); 23.40 (message group)	50% males (control); 55% males (message group)	University students	A correcting message (targeting the illusion of control), every 15th game played	Pop-up with the word “Pause”	Behavior and Cognition	Behavior: Number of games played; Cognition: Erroneous beliefs	None
Ginley et al. (53)	Lab/RCT	154	22.7	Approximately 60% females	University students	Periodic warning messages	“Press ok to continue”	Behavior	Total number of spins	1 week follow-up
Harris and Parke (32)	Lab/RCT	26 (30)	23.08/30	18 males; 12 females	Gamblers playing on EGMs within 6 months prior to participation	Self-appraisal message	Pre pop-up intervention	Behavior	Betting speed, average stake size and betting intensity	None

*NR, not reported; Lab, laboratory study; RCT, randomized controlled trial; RG, responsible gambling; EGM, Electronic Gaming Machine*.

*Explanations of measures: Keeping track of play usage=amount spent, time played, spins played; Estimation accuracy=amount spent, time played, spins played; Player experience=dissociation, enjoyment, annoyance/frustration*.

**Table 4:** Study characteristics, studies H-M.

**References**	**Study design**	**Participants (N at start of trial in parenthesis)**	**Mean age**	**Sex**	**Inclusion criteria**	**Type of intervention in experimental condition**	**Type of intervention in control condition**	**Outcome**	**Measures**	**Follow-up**
Harris et al. (65)	Lab/RCT	65 (70)	31.14	53/70 males	Regular gamblers	Emotive vs. informative pop-ups	No pop-up	Behavior and Cognition	Behavior: Reaction time, response inhibition; Cognition: Arousal, valence and dissociation; IST choice latency, IST balls sampled, IST p-correct and MCQ k-value	None
Hollingshead et al. (47)	Lab/RCT	98	50.25	42 males	Gamblers at their respective casinos to play slots but had not yet gambled	RG educational information provided in advance of a RG-related decision	RG information not tied to RG decision making	Cognition	Desire to gamble, self-reported dissociation during play, future limit setting intentions	None
Jardin and Wulfert (44)	Lab/RCT	80	44	60 males; 20 females	High-frequency gamblers	Accurate vs. inaccurate vs. neutral pop-up	No pop-up	Behavior	Total bet amount, number of trials played	None
McGivern et al. (37)	Lab/RCT	45	NR	19 males; 24 females; 2 undisclosed	Casual gamblers	Expenditure-specific warning messages	“Press ok to continue”	Behavior	Amount wagered during “open bets”	None
Mizerski et al. (66)	Naturalistic/RCT	831	NR	NR	University students	Strong vs. weak message	No pop-up	Behavior	Number of bets, bet amount, length of gambling session	None
Monaghan and Blaszczynski (39)	Lab/RCT	92	19.3	75% females	University students	Dynamic standard message	Static message	Cognition	Free recall, cued recall, recall accuracy, recollection confidence	None
Monaghan and Blaszczynski (33)^a^	Lab/RCT	127	20.3	76.4% males	University students	Dynamic: Informative vs. self-appraisal	Static pop-up without content	Behavior and Cognition	Behavior: Impact on real EGM-play, influence on session length, within-session behavior; Cognition: Awareness of time, disruption, recall, within-session thoughts	2 weeks follow-up

*IST, Information Sampling Task; MCQ, Monetary Choice Questionaire*.

*Explanation of variables*.

a *Study 1/2*.

**Table 5:** Study characteristics, studies M-W.

**References**	**Study design**	**Participants (N at start of trial in parenthesis)**	**Mean age**	**Sex**	**Inclusion criteria**	**Type of intervention in experimental condition**	**Type of intervention in control condition**	**Outcome**	**Measures**	**Follow-up**
Monaghan and Blaszczynski (33)^a^	Naturalistic/RCT	124	44.1	71.8% males	Regular gamblers	Dynamic: Informative vs. self-appraisal	Static pop-up without content	Behavior and Cognition	Behavior: Impact on real EGM-play, influence on session length, likelihood of taking a break, likelihood of cashing out prize, likelihood of leaving, within-session behavior; Cognition: Influence awareness, recall, within-session thoughts, disruption	2 weeks follow-up
Rockloff et al. (48)	Lab/RCT	107	NR	45 males; 62 females	Casual gamblers	Relevant pop-up messag	No pop-up	Behavior and Cognition	Behavior: Average bets, bets pr. minute, total trials, losses, skin conductance (SC); Cognition: Enjoyment	None
Stewart and Wohl (40)	Lab/RCT	59	20.76	43 males; 16 females	University students	Monetary limit pop-up	No pop-up	Behavior and Cognition	Behavior: Adherence to self-proclaimed monetary limits; Cognition: Dissociation, craving to continue	None
Tabri et al. (38)	Lab/RCT	88	55.13	54.5% females	Community gamblers	Monetary limit pop-ups: approaching limit vs. 90% of limit vs. 70% of limit	No pop-up	Behavior	Percentage of players who stopped gambling before reaching monetary limit	None
Wohl et al. (41)^b^^)^	Lab/RCT	72	19.69	70.8% females	University students	Monetary limit pop-up	No pop-up	Behavior and Cognition	Behavior: Adherence to pre-set monetary limit; Cognition: Erroneous cognition, limit detection	None
Wohl et al. (67)^c^	Lab/RCT	56	20.38	19 males; 37 females	Casual gamblers	HCI and PSD inspired pop-up(s)	Standard monetary limit pop-up	Behavior and Cognition	Behavior: Adherence to pre-set monetary limit, engagement with pop-up tool; Cognition: Dissociation	None

*HCI, Human Computer Interaction; PSD, Persuasive Systems Design*.

*Explanations of measures: Influence awareness=money spent, time, estimation of prize, understanding play, estimation of win/loss*.

a *Study 2/2*.

b *Study 1/2*.

c *Study 2/2*.

### Risk of Bias

Risk of bias of the included studies was assessed using the Evidence Project Risk of Bias Tool (54). This instrument was used by two authors independently, rating each of the 18 studies. Disagreements were resolved by consulting the third author. Of the 18 studies included, one met the criteria of cohort. Seventeen studies had a control or a comparison group, whereas one was based on a pre-post measurement design. In total, six studies reported data both pre- and post-pop-up intervention. All except two studies scored “yes” on “random assignment of participants to the intervention”; the two that did not were rated “not applicable” as their datasets were anonymous and provided by a real-world online gambling site. Two studies had random selection of participants of assessment, whereas the other 16 consisted of gamblers. Of the two studies containing follow up intervention, one met the criteria of retaining at least 80% of the participants.

All but two studies had comparison groups equivalent on socio-demographics (both rated “not applicable”). Lastly, two studies reported comparison groups equivalent on outcome measures at baseline, whereas the others were categorized as “not reported.” A calculation of inter-rater reliability of risk of bias yielded a Cohen's Kappa of 0.96 which according to Landis and Koch (68) is regarded as a perfect agreement. The risk of bias in the included studies is shown in Table 6.

**Table 6:** Evaluation of risk of bias in the individual studies.

**Study**	**Cohort**	**Control or comparison group**	**Pre-post intervention data**	**Random assignment of participants to the intervention**	**Random selection of participants for assessment**	**Follow-up rate of 80% or more**	**Comparison groups equivalent on socio- demographics**	**Comparison groups equivalent on outcome measures at baseline**
Auer et al.	No	Yes	Yes	NA	No	NA	NA	NR
Auer and Griffiths	No	Yes	Yes	NA	No	NA	NA	NR
Byrne and Russell	No	Yes	Yes	Yes	Yes	NA	Yes	Yes
Cloutier et al.	No	Yes	Yes	Yes	No	NA	Yes	NR
Ginley et al.	Yes	Yes	No	Yes	No	No	Yes	NR
Harris and Parke	No	No	Yes	Yes	No	NA	Yes	NR
Harris et al.	No	Yes	No	Yes	No	NA	Yes	NR
Hollingshead et al.	No	Yes	No	Yes	No	NA	Yes	NR
Jardin and Wulfert	No	Yes	No	Yes	No	NA	Yes	NR
McGivern et al.	No	Yes	No	Yes	No	NA	Yes	NR
Mizerski et al.	No	Yes	No	Yes	No	NA	Yes	NR
Monaghan and Blaszczynski^a^	No	Yes	No	Yes	No	NA	Yes	NR
Monaghan and Blaszczynski^b^	No	Yes	Yes	Yes	No	Yes^c^	Yes	Yes
Rockloff et al.	No	Yes	No	Yes	Yes	NA	Yes	NR
Stewart and Wohl	No	Yes	No	Yes	No	NA	Yes	NR
Tabri et al.	No	Yes	No	Yes	No	NA	Yes	NR
Wohl et al.^d^	No	Yes	No	Yes	No	NA	Yes	NR
Wohl et al.^e^	No	Yes	No	Yes	No	NA	Yes	NR
Auer et al.	No	Yes	Yes	NA	No	NA	NA	NR

*NR, not reported; NA, not applicable*.

a *Monaghan and Blaszczynski (39)*.

b *Monaghan and Blaszczynski (33)*.

c *Only study 1 fulfilled this criteria*.

d *Wohl et al. (41)*.

e *Wohl et al. (67)*.

### Synthesized Findings

#### Cognitive Measures

**Figure 2:**
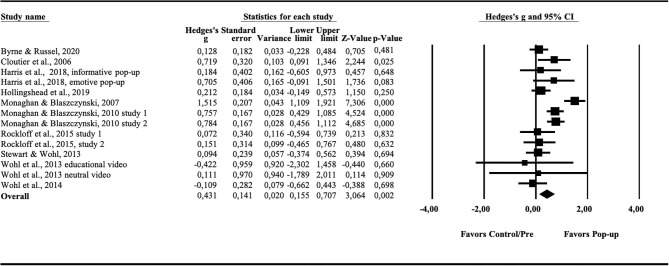
Forest plot showing the effect (Hedges' g) of pop-up messages on cognitive measures.

**Figure 3:**
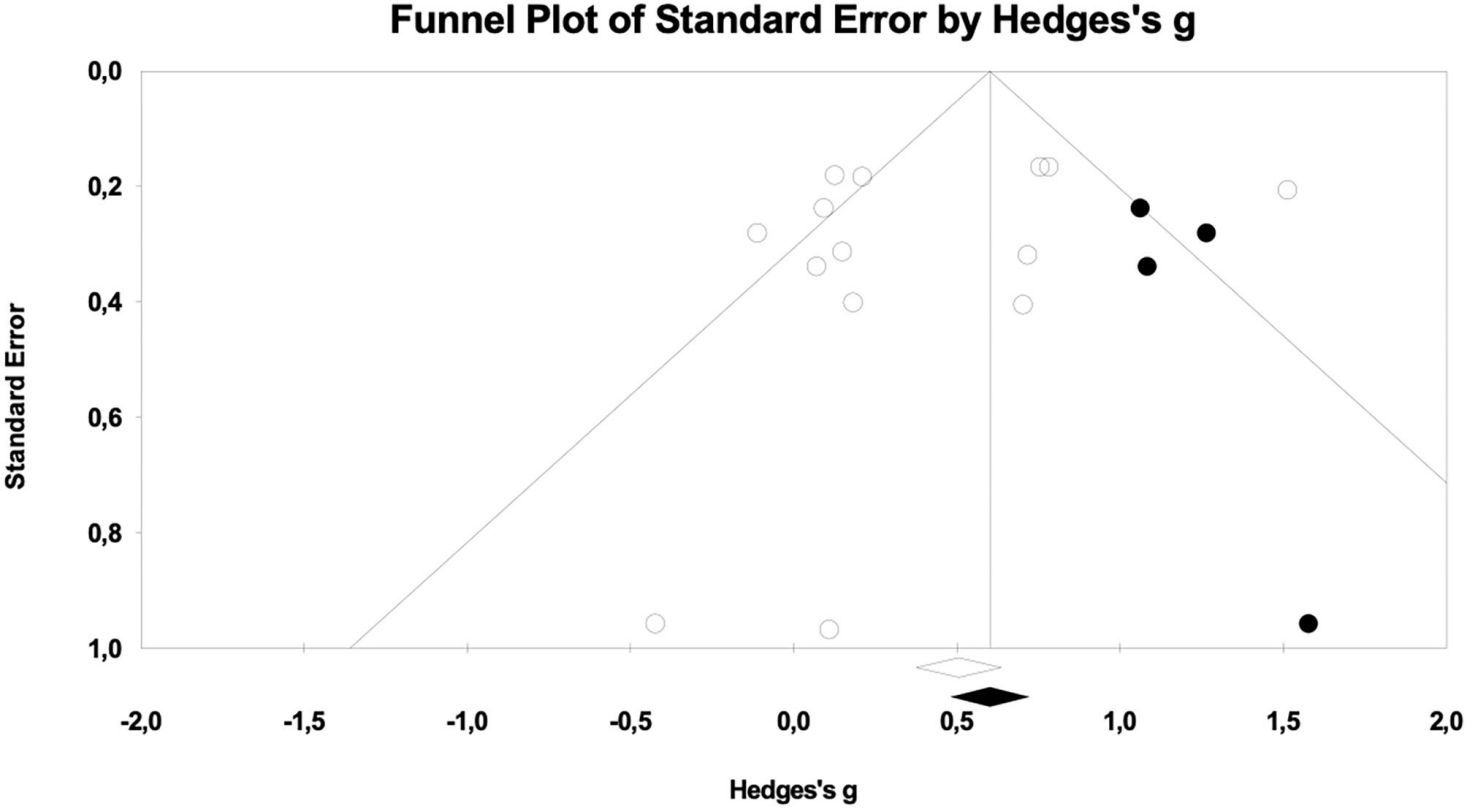
Funnel plot from the meta-analysis of cognitive measures, adjusted using Duval and Tweedie's trim and fill.

Results for cognitive measures after pop-up intervention during gambling showed an overall effect size of *g* = 0.413 (95% CI = 0.155–0.707), *p* < 0.01 (see Figure 2). Cochrane's *Q* was 48.63 (df = 13), *p* < 0.01) and the *I*^2^ was 73.27. No subgroup analysis was performed due to lack of studies including non-gamblers (*k* = 2) and *in situ* trials (*k* = 1). In order to investigate whether the present findings were influenced by publication bias, a funnel plot was drawn. The funnel plot was not entirely symmetrical (see Figure 3), suggesting a lack of potential studies to the right of the distribution. The Duval and Tweedie s “trim and fill” procedure provided an adjusted effect size g = 0.578 (95% CI = 0.325–0.830, *p* < 0.01). Orwin's fail-safe N showed that 22 studies with zero effect would be needed to bring the overall effect size down to a trivial level (g = 0.20).

#### Behavioral Measures

**Figure 4:**
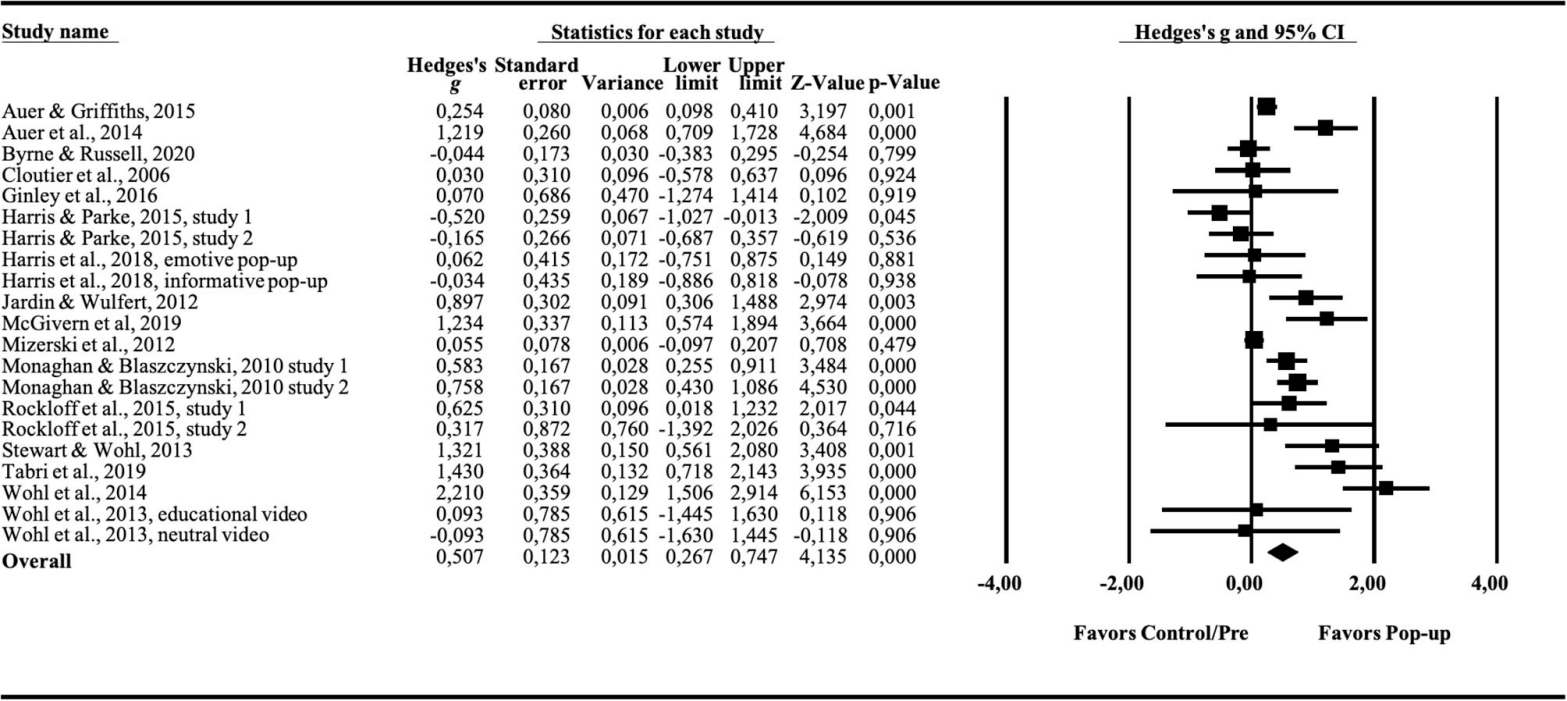
Forest plot showing the effect (Hedges' g) of pop-up messages on behavioral measures.

**Figure 5:**
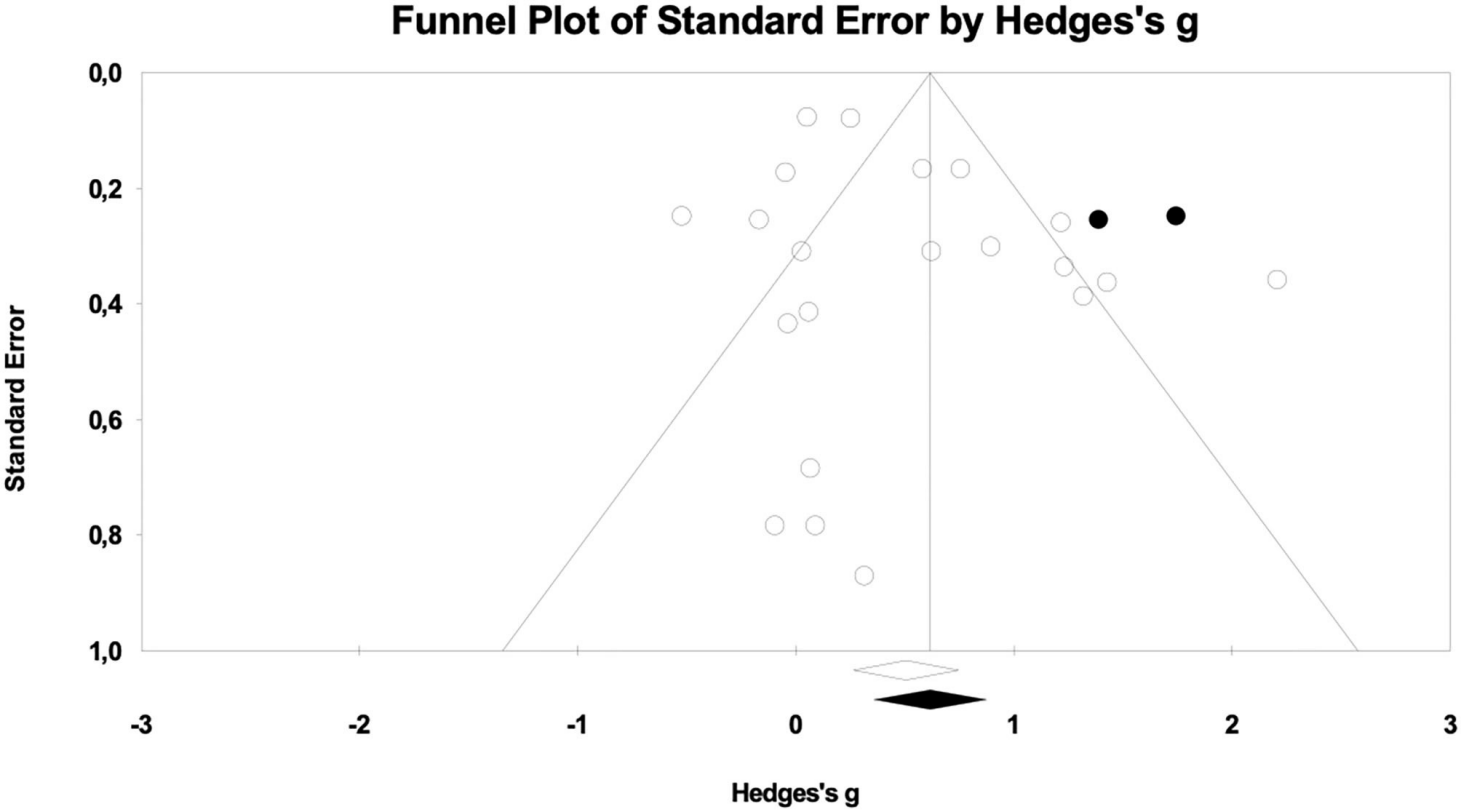
Funnel plot from the meta-analysis of behavioral measures, adjusted using Duval and Tweedie's trim and fill.

The overall effect size for behavioral measures was *g* = 0.507 (95% CI = 0.267–0.747, *p* < 0.01; see forest plot, Figure 4). Cochrane's *Q* was 109.84 (df = 20), *p* < 0.5, and *I*^2^ = 81.97, suggesting significant heterogeneity. Four studies (*k* = 4) were done in a naturalistic setting and 17 were performed in a laboratory setting, and thus, a subgroup analysis was performed. The effect size difference turned out not significant (*Q*_bet_ = 0.010, df = 1, *p* = 0.919). No subgroup analysis was performed due to the lack of studies containing non-gamblers (*k* = 1). To detect potential publication bias, a funnel plot was drawn. The plot was not symmetrical (see Figure 5), and indicated lack of potential studies to the right of the distribution. Hence, Duwal and Tweedie's “trim and fill” procedure was conducted, providing an adjusted effect size of 0.616 (95% CI = 0.359–0.872, *p* < 0.01). Orwin's fail-safe *N* showed that the number of missing studies with zero effect needed to bring Hedges' *g* below 0.20, was 12.

## Discussion

The aim of the present meta-analysis was to investigate the effect of gambling pop-up message interventions on behavioral and cognitive outcomes at first exposure and at follow-up, as well as to investigate the potential moderating effect of study setting and sample characteristics. The meta-analysis of behavioral measures demonstrated a significant effect amounting to a medium effect size in accordance with Cohen (63), indicating that RG pop-up message interventions have a substantial impact on participants' gambling behavior. The heterogeneity was significant, reflecting true differences in effect size across studies (59). Consequently, a subgroup analysis was conducted. However, no significant difference between effects from laboratory settings and naturalistic settings was found. Orwin's fail-safe *N* demonstrated that the total number of studies needed to bring the effect size down to a trivial level (*g* = 0.20), was 12, which suggest some stability of the findings.

A total of five studies provided effect sizes above 1.00 (35, 37, 38, 38, 40, 67). When looking at the pop-ups from these studies, they all appeared to be personalized in terms of gambling behavior [e.g., “*You have reached your preset limit”*; (40)], whereas pop-ups of studies with no effect appeared to be more generic [e.g., “*No matter how you play, you cannot influence the outcome of the game”*; (64)]. Regarding effects in the three real world studies, the first study showed that when being informed of having played 1,000 slot games, 45 of 4,205 sessions ended compared to 5 of 4,220 sessions before the pop-up had been introduced by the gaming operator (35). The second study found that 169 of 11,878 sessions ended following an enhanced pop-up message compared to 75 of 11,232 sessions with a simple pop-up message (36). The third study reported a mean number of spins and amount wagered of 15.8 and $17.70 in the strong pop-up message condition, respectively, whereas the corresponding values in the control condition were 18.1 and $24.90 (66).

A significant and positive effect was also found for the cognitive measures, reflecting a medium effect size. Also here, the heterogeneity was significant. No subgroup analysis was conducted, as there were too few studies in the smallest of the subgroups. Orwin's fail-safe *N* demonstrated that the total number of studies with zero effect needed to bring the overall effect size down to a trivial level (g = 0.20) was 22, indicating that the findings are stable.

The gamblers vs. non-gambler moderator, was chosen as previous studies have shown that gamblers diverge from non-gamblers on some gambling-related behaviors and cognitions compared to non-gamblers (69). For instance, it has been found that gamblers discount probabilistic rewards less steeply than non-gamblers (70), that laboratory trials with student populations yield larger effects than with non-student populations (71), and that gamblers take larger monetary risks during roulette play than non-gamblers (72). The second moderator (laboratory vs. real gambling settings) was emphasized as there might be ecological challenges associated with laboratory studies such as the laboratory cubicle-casino ambiance variance and the absence of direct or personal monetary risk or loss (71, 73). The fact that this moderator turned out non-significant may be due to the relatively small amount of studies included in naturalistic settings. It may also reflect that other potential moderators, not identified by the authors, could explain significant proportions of the heterogeneity. The present analyses of heterogeneity could therefore have benefited from either different or simply more moderators.

### Implications for RG Practices

The results of the present meta-analysis show that pop-up messaging appear to be an effective RG tool, as it seemed to reduce possible harmful gambling behaviors and cognitions. As previous studies have indicated, the intervention of a pop-up message reduces, amongst other things, the total amount wagered, spins per minute, and irrational beliefs. Within the framework of responsible gambling in both the Reno Model and the Public Health Model, these results are encouraging, as it complies with some of the models' central tenets: the emphasis on a scientific approach in the development of measures to promote responsible gambling and at the same time, reducing gambling-related harm. Furthermore, the non-intrusive and non-restrictive nature of pop-up messages coincides with three central Reno Model principles: (i) the opportunity to gamble without intrusion, (ii) the maintenance of personal control (as opposed to, for example, reduced casino opening hours), and (iii) the opportunity to gamble in an unrestricted, but informed manner. In addition to this, the result of the present meta-analyses provides further evidence of the suggestion that gamblers gamble more responsibly (e.g., fewer spins, less money wagered, less time spent playing, increased limit-adherence) when informed about the nature of the game they are playing (41, 44, 74). Finally, the relative non-intrusive nature of a pop-up message could help facilitate increased collaboration between different stakeholders involved in gambling, as emphasized in the Reno Model (17). This is further attested to, because major online gambling sites already have implemented pop-up messages as an RG feature (35, 36).

Although there is some support suggesting that RG pop-up messages influence behavior of a small number of people playing for a long time (1,000 or more spins on a virtual roulette game) (35), the failure to identify a sufficient number of studies containing problem gamblers, makes it difficult to assess the effect of pop-up messages on gamblers who already suffer from gambling-related problems, and therefore fails to shed any new light on its effectiveness in mitigating problematic gambling among problem gamblers. However, the effect it has on gambling behavior of casual/regular gamblers, contributes (at least short-term) to a reduction of gambling harm, which in turn promotes a public health perspective of safe gambling (17). As gambling becomes increasingly digitalized, pop-up messaging appears to represent an accessible and cost-effective way to attenuate excessive gambling behavior and to modify gambling-related cognitions.

### Limitations and Future Directions

The present meta-analysis has several limitations that should be noted. One limitation concerns the risk of bias of the included studies, with several studies failing to pass some of the criteria of the assessment tool used (54). Most of the studies lacked one or more of the following: (i) a cohort (pre and post data on the same subjects), (ii) random selection of participants for assessment, (iii) follow-up assessments, and (iv) did not report whether there was equivalency on outcome measures at baseline for comparison groups. Only two studies (33, 53) included follow-up data. The lack of follow-up data in the included studies makes it difficult to draw conclusions about the long-term effects of pop-up interventions on gambling behavior and cognition. Furthermore, the absence of random sampling and equivalency on outcome measures at baseline for comparison groups, should be considered a limitation, as it can limit the generalizability of the results (59).

Only two of the included studies (35, 36) evaluated gamblers in their real-life gambling environments (i.e., actual gambling with players spending their own money). It is therefore important to be cognizant of the ecological validity of the findings from the majority of studies included in the present meta-analysis. Even though it has been shown that certain types of rewards (i.e., the possibility to win via raffle or lottery tickets) can be as effective as immediate monetary rewards (75–77), there is still reason to question the true ecological validity and generalizability of such trials, as, for example, the absence of risking one's own money in a gambling situation, has been shown to increase spending in gamblers (78). Other limitations related to studying gambling behavior in laboratory settings are that such settings often lack aspects present in real-life gambling such as variety of gambling motives, ability to choose between different games, playing games in different ways, and the distinct milieu of gambling venues (79). Therefore, more studies on the effects of pop-ups should be conducted in real world gambling contexts with real gamblers in real time [like those of Auer et al. (35) and Auer and Griffiths (36)]. Another limitation of the present meta-analysis is that outcomes subsumed as cognitive outcomes varied significantly. However, the limited number of studies prevented meta-analyses of more narrow outcomes and constructs.

One area for future research concerns the long-term effects of pop-ups. It can be argued that the main aim of pop-ups is to change behaviors and cognitions in the specific context of a gambling session. Consequently, it would be of interest to investigate whether repeated exposure to pop-ups during gambling can cause long-lasting and robust changes in gambling behaviors and cognitions or not. This should be addressed in future research. In addition to this, the authors of the present review echo other researchers' call for further investigation into the possible habituation of warning messages (31, 52). The effect of pop-ups in specific vulnerable populations (e.g., problem gamblers) should also be addressed in future studies.

The comparison condition of the included studies differed across studies. Some comprised a non-pop-up intervention, whereas other comprised a pause or neutral or irrelevant pop-up messages. This makes it difficult to conclude whether some of the effects are attributable to the presence of a pop-up in itself, or whether the effects were contingent on the specific form, placement, duration, or content of the pop-up. Given that the experimental interventions differed in message content (e.g., limit reminders, self-appraisal feedback, personalized feedback), future studies and meta-analyses are advised also to further investigate the effects of differences in message content. The present meta-analysis did not investigate the effects of pauses *per se* during gambling, and pure pauses comprised the control condition in several studies. Therefore, the present meta-analysis does not provide information about the effect of pauses on gambling cognitions and behavior. We thus recommend that future studies and reviews systematically investigate the effects of pauses, preferably by experimental designs in real-world settings.

## Conclusions

The present meta-analysis examined the efficacy of RG pop-up messages on gambling behaviors and cognitions. The results showed that RG pop-up messages had a moderate effect on gambling behaviors and cognitions, using interventions which should be considered as highly cost-effective. The present meta-analysis is of importance, as it is the first meta-analyses on the efficacy of pop-up messages on gambling behaviors and cognitions [although narrative literature reviews have been previously conducted (34, 80, 81)]. As such, the meta-analysis contributes to the literature by filling an important gap in knowledge of the efficacy of pop-up messages as a tool to promote responsible gambling. The findings imply that there are benefits to using pop-up messages to promote responsible gambling, although caution should be exercised in terms of generalizability to real-world gambling settings, hence more studies in such contexts should be conducted.

## Data Availability Statement

The raw data supporting the conclusions of this article will be made available by the authors, without undue reservation.

## Ethics Statement

As only secondary data were analyzed the study was exempted from approval by Regional Committees for Medical and Health Research Ethics.

## Author Contributions

SP assisted by BB, JS, and AB: conceptualization. BB, JS, and AB: literature search and study coding. BB, JS, and AB assisted by SP: data analysis. BB, JS, AB, and SP: drafting the manuscript. EE, TL, and MG: critically revising the original manuscript and the revised version. All authors contributed to finalizing the paper and approved the submitted version.

## Conflict of Interest

MG's university currently receives research funding from Norsk Tipping (the gambling operator owned by the Norwegian Government). MG has also received funding for a number of research projects in the area of gambling education for young people, social responsibility in gambling and gambling treatment from Gamble Aware (formerly the Responsible Gambling Trust), a charitable body which funds its research program based on donations from the gambling industry. MG regularly undertakes consultancy for various gaming companies in the area of social responsibility in gambling. The remaining authors declare that the research was conducted in the absence of any commercial or financial relationships that could be construed as a potential conflict of interest.
